# Erlotinib hydro­chloride: an anti­cancer agent

**DOI:** 10.1107/S1600536808011707

**Published:** 2008-04-30

**Authors:** S. Selvanayagam, B. Sridhar, K. Ravikumar

**Affiliations:** a Department of Physics, Kalasalingam University, Krishnankoil 626 190, India; b Laboratory of X-ray Crystallography, Indian Institute of Chemical Technology, Hyderabad 500 007, India

## Abstract

In the cation of the title compound, C_22_H_24_N_3_O_4_^+^·Cl^−^, an active ingredient of the anti­cancer drug also known as Tarceva, the quinazoline ring system is planar within 0.044 (3) Å. The dihedral angle formed by the mean planes of the two six-membered quinazoline rings is 3.2 (1)°. Both N-bound H atoms participate in N—H⋯Cl bonds, which link the ions into infinite chains running along the *b* axis. C—H⋯O inter­actions involving neighboring cations provide additional stabilization of these aggregates.

## Related literature

For related literature, see: Herbst *et al.* (2005 ▶); Minna & Dowell (2005 ▶); Li *et al.* (2007 ▶); Xia (2005 ▶). For bond-length data, see: Allen *et al.* (1987 ▶).
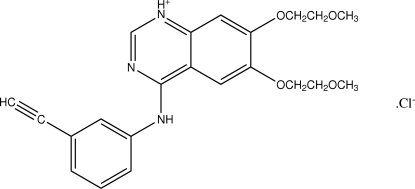


## Experimental

### 

#### Crystal data

C_22_H_24_N_3_O_4_^+^·Cl^−^
*M* *_r_* = 429.89
Monoclinic, 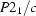

*a* = 14.5351 (15) Å
*b* = 18.4863 (19) Å
*c* = 8.1222 (8) Å
β = 102.966 (2)°
*V* = 2126.8 (4) Å^3^
*Z* = 4
Mo *K*α radiation
μ = 0.21 mm^−1^
*T* = 293 (2) K
0.26 × 0.22 × 0.20 mm

#### Data collection

Bruker APEX area-detector diffractometer
Absorption correction: none
24377 measured reflections
5001 independent reflections
3649 reflections with *I* > 2σ(*I*)
*R*_int_ = 0.050

#### Refinement

*R*[*F* ^2^ > 2σ(*F* ^2^)] = 0.073
*wR*(*F* ^2^) = 0.151
*S* = 1.16
5001 reflections
275 parameters
H atoms treated by a mixture of independent and constrained refinement
Δρ_max_ = 0.33 e Å^−3^
Δρ_min_ = −0.25 e Å^−3^

### 

Data collection: *SMART* (Bruker, 2001 ▶); cell refinement: *SAINT* (Bruker, 2001 ▶); data reduction: *SAINT*; program(s) used to solve structure: *SHELXS97* (Sheldrick, 2008 ▶); program(s) used to refine structure: *SHELXL97* (Sheldrick, 2008 ▶); molecular graphics: *ORTEP-3* (Farrugia, 1997 ▶) and *PLATON* (Spek, 2003 ▶); software used to prepare material for publication: *SHELXL97* and *PARST* (Nardelli, 1995 ▶).

## Supplementary Material

Crystal structure: contains datablocks I, global. DOI: 10.1107/S1600536808011707/ya2075sup1.cif

Structure factors: contains datablocks I. DOI: 10.1107/S1600536808011707/ya2075Isup2.hkl

Additional supplementary materials:  crystallographic information; 3D view; checkCIF report

## Figures and Tables

**Table 1:** Hydrogen-bond geometry (Å, °)

*D*—H⋯*A*	*D*—H	H⋯*A*	*D*⋯*A*	*D*—H⋯*A*
N3—H3⋯Cl1	0.86	2.46	3.277 (2)	160
N1—H*N*1⋯Cl1^i^	0.86	2.23	3.066 (2)	165
C1—H1⋯O4^i^	0.93	2.46	3.372 (3)	167

Symmetry code: (i) 
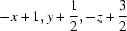
.

**Fig. 1.**
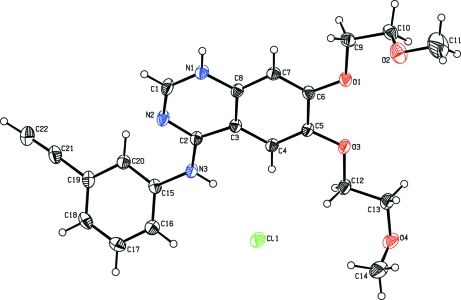
The structure and atom-numbering scheme for (I); displacement ellipsoids are drawn at the 30% probability level and H atoms are shown as small spheres of arbitrary radius.

**Fig. 2.**
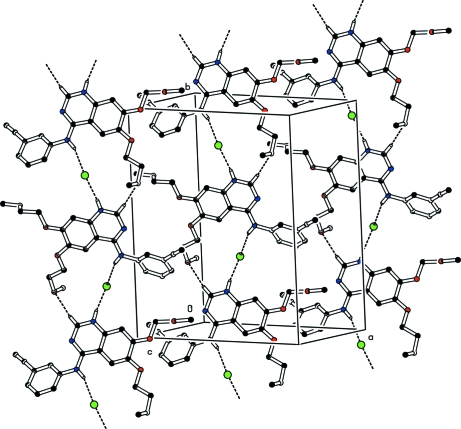
Molecular packing of (I) viewed along the c axis; H-bonds are shown as dashed lines; H atoms, not involved in H-bonds, have been omitted.

## supplementary crystallographic information

### Comment

Erlotinib hydrochloride, (I), also known under its tradename Tarceva, is a potent reversible epidermal growth factor receptor tyrosine kinase inhibitor with single agent activity in patients with non-small lung cancer, pancreatic cancer and several other types of cancer (Herbst *et al.*, 2005). It is the first drug to demonstrate an increase in survival in phase III trials in patients with advanced non-small cell lung cancer (Minna & Dowell, 2005). It has recently been shown to be potent inhibitor of JAK2 V617F activity; JAK2 V617F is a mutant of tyrosine kinase JAK2 (Li *et al.*, 2007). As no crystal structure of the title compound has yet been published, we have undertaken the single-crystal X-ray diffraction study and report here its results.

The X-ray study confirmed the molecular structure and atomic connectivity for (I), as illustrated in Fig. 1. The C21—C22 bond length [1.170 (4) Å] is consistent with its acetylenic character, as evidenced by literature value of 1.174 (11) Å (see Allen *et al.*, 1987). The geometry of the quinazoline ring system is comparable to that in the reported related structure (Xia, 2005).

The bicyclic system is effectively planar with a maximum deviation of 0.044 (3) Å for the C1 atom. The dihedral angle formed by the mean planes of two six membered rings of quinazoline moiety is 3.2 (1)°. The benzene ring C15—C20 and its attached ethynyl group are coplanar with a maximum deviation -0.011 (3) Å for the C20 atom. The dihedral angle between this ring and quinazoline ring system is 34.4 (1)°. The short contacts H3···H4 (2.12 Å) and H7···H9A (2.14 Å) result in substantial widening of the C2—N3—C15 and C6—O1—C9 bond angles [127.4 (2)° and 117.1 (2)°, respectively].

Both N-bound H atoms (HN1 and H3) participate in H-bonds with the Cl1 anion (Table 2). These bonds link cations and anions into the infinite chains running along the *b* axis of the crystal. The C1—H1···O4 interactions involving neighboring cations provide additional stabilization for these chains (Fig. 2).

### Experimental

In order to obtain crystals suitable for X-ray study, commercially available erlotinib hydrochloride was dissolved in a methanol-water solution (90:10v/v); the solvents were then allowed to evaporate slowly.

### Refinement

The acetylenic H22 atom was located in a difference Fourier map and refined isotropically [C22—H22 0.95 (3) Å]; all other H atoms were positioned geometrically with C—H distances of 0.93–0.97 Å, N—H 0.86 Å and were included in the refinement in the riding motion approximation with *U*_iso_= 1.5*U*_eq_(C) for methyl H and 1.2*U*_eq_ for all other H atoms.

### Crystal data

**Table:**

C_22_H_24_N_3_O_4_^+^·Cl^–^	*F*_000_ = 904
*M**_r_* = 429.89	*D*_x_ = 1.343 Mg m^−^^3^
Monoclinic, *P*2_1_/*c*	Mo *K*α radiation λ = 0.71073 Å
Hall symbol: -P 2ybc	Cell parameters from 9448 reflections
*a* = 14.5351 (15) Å	θ = 2.2–23.4º
*b* = 18.4863 (19) Å	µ = 0.21 mm^−^^1^
*c* = 8.1222 (8) Å	*T* = 293 (2) K
β = 102.966 (2)º	Block, colourless
*V* = 2126.8 (4) Å^3^	0.26 × 0.22 × 0.20 mm
*Z* = 4	

### Data collection

**Table:**

Bruker APEX area-detector diffractometer	3649 reflections with *I* > 2σ(*I*)
Radiation source: fine-focus sealed tube	*R*_int_ = 0.050
Monochromator: graphite	θ_max_ = 28.0º
*T* = 293(2) K	θ_min_ = 1.8º
ω scans	*h* = −18→19
Absorption correction: none	*k* = −24→24
24377 measured reflections	*l* = −10→10
5001 independent reflections	

### Refinement

**Table:**

Refinement on *F*^2^	Secondary atom site location: difference Fourier map
Least-squares matrix: full	Hydrogen site location: inferred from neighbouring sites
*R*[*F*^2^ > 2σ(*F*^2^)] = 0.073	H atoms treated by a mixture of independent and constrained refinement
*wR*(*F*^2^) = 0.151	*w* = 1/[σ^2^(*F*_o_^2^) + (0.0551*P*)^2^ + 0.7563*P*] where *P* = (*F*_o_^2^ + 2*F*_c_^2^)/3
*S* = 1.16	(Δ/σ)_max_ = 0.001
5001 reflections	Δρ_max_ = 0.33 e Å^−^^3^
275 parameters	Δρ_min_ = −0.25 e Å^−^^3^
Primary atom site location: structure-invariant direct methods	Extinction correction: none

### Special details

**Table:**

Geometry. All e.s.d.'s (except the e.s.d. in the dihedral angle between two l.s. planes) are estimated using the full covariance matrix. The cell e.s.d.'s are taken into account individually in the estimation of e.s.d.'s in distances, angles and torsion angles; correlations between e.s.d.'s in cell parameters are only used when they are defined by crystal symmetry. An approximate (isotropic) treatment of cell e.s.d.'s is used for estimating e.s.d.'s involving l.s. planes.
Refinement. Refinement of *F*^2^ against ALL reflections. The weighted *R*-factor *wR* and goodness of fit *S* are based on *F*^2^, conventional *R*-factors *R* are based on *F*, with *F* set to zero for negative *F*^2^. The threshold expression of *F*^2^ > σ(*F*^2^) is used only for calculating *R*-factors(gt) *etc*. and is not relevant to the choice of reflections for refinement. *R*-factors based on *F*^2^ are statistically about twice as large as those based on *F*, and *R*- factors based on ALL data will be even larger.

### Fractional atomic coordinates and isotropic or equivalent isotropic displacement parameters (Å^2^)

**Table:**

	*x*	*y*	*z*	*U*_iso_*/*U*_eq_	
Cl1	0.50200 (5)	0.34427 (3)	0.62635 (9)	0.0469 (2)	
O1	0.27725 (11)	0.61695 (9)	0.9544 (2)	0.0395 (4)	
O2	0.10485 (14)	0.70220 (13)	0.9170 (3)	0.0648 (6)	
O3	0.29876 (12)	0.48732 (9)	0.8521 (2)	0.0417 (4)	
O4	0.20059 (13)	0.31485 (10)	0.6875 (3)	0.0514 (5)	
N1	0.57019 (14)	0.69146 (10)	0.8273 (3)	0.0395 (5)	
HN1	0.5603	0.7355	0.8527	0.047*	
N2	0.67238 (14)	0.60941 (11)	0.7423 (3)	0.0408 (5)	
N3	0.63506 (13)	0.48880 (10)	0.7058 (3)	0.0350 (5)	
H3	0.5945	0.4550	0.7065	0.042*	
C1	0.64787 (18)	0.67467 (13)	0.7780 (4)	0.0435 (7)	
H1	0.6887	0.7123	0.7678	0.052*	
C2	0.61060 (16)	0.55508 (12)	0.7464 (3)	0.0318 (5)	
C3	0.52182 (16)	0.56803 (12)	0.7918 (3)	0.0310 (5)	
C4	0.45169 (16)	0.51536 (12)	0.7961 (3)	0.0321 (5)	
H4	0.4604	0.4681	0.7637	0.039*	
C5	0.37123 (16)	0.53333 (13)	0.8474 (3)	0.0324 (5)	
C6	0.35795 (16)	0.60529 (13)	0.9019 (3)	0.0326 (5)	
C7	0.42426 (17)	0.65734 (12)	0.8963 (3)	0.0346 (5)	
H7	0.4158	0.7044	0.9305	0.042*	
C8	0.50471 (16)	0.63882 (12)	0.8385 (3)	0.0320 (5)	
C9	0.26703 (18)	0.68616 (14)	1.0300 (4)	0.0417 (6)	
H9A	0.2742	0.7248	0.9530	0.050*	
H9B	0.3151	0.6919	1.1333	0.050*	
C10	0.17127 (19)	0.68926 (15)	1.0675 (4)	0.0448 (6)	
H10A	0.1574	0.6439	1.1166	0.054*	
H10B	0.1689	0.7277	1.1478	0.054*	
C11	0.0123 (3)	0.7093 (3)	0.9450 (7)	0.1109 (16)	
H11A	−0.0314	0.7178	0.8392	0.166*	
H11B	0.0106	0.7492	1.0197	0.166*	
H11C	−0.0047	0.6656	0.9949	0.166*	
C12	0.30023 (17)	0.41876 (13)	0.7695 (4)	0.0422 (6)	
H12A	0.3512	0.3889	0.8318	0.051*	
H12B	0.3093	0.4256	0.6558	0.051*	
C13	0.2062 (2)	0.38355 (15)	0.7650 (4)	0.0510 (7)	
H13A	0.1974	0.3784	0.8793	0.061*	
H13B	0.1560	0.4142	0.7029	0.061*	
C14	0.1801 (3)	0.3188 (2)	0.5094 (5)	0.0765 (11)	
H14A	0.1774	0.2709	0.4631	0.115*	
H14B	0.2286	0.3460	0.4740	0.115*	
H14C	0.1203	0.3423	0.4699	0.115*	
C15	0.72087 (16)	0.46773 (12)	0.6616 (3)	0.0327 (5)	
C16	0.71529 (18)	0.41268 (14)	0.5436 (3)	0.0396 (6)	
H16	0.6575	0.3912	0.4968	0.048*	
C17	0.7960 (2)	0.38986 (16)	0.4959 (4)	0.0474 (7)	
H17	0.7922	0.3529	0.4169	0.057*	
C18	0.88191 (19)	0.42120 (15)	0.5641 (4)	0.0473 (7)	
H18	0.9356	0.4060	0.5296	0.057*	
C19	0.88874 (17)	0.47541 (13)	0.6842 (3)	0.0395 (6)	
C20	0.80763 (17)	0.49842 (13)	0.7347 (3)	0.0371 (6)	
H20	0.8118	0.5341	0.8168	0.045*	
C21	0.9793 (2)	0.50773 (16)	0.7563 (4)	0.0490 (7)	
C22	1.0541 (2)	0.5325 (2)	0.8100 (5)	0.0653 (9)	
H22	1.115 (2)	0.5534 (16)	0.849 (4)	0.063 (9)*	

### Atomic displacement parameters (Å^2^)

**Table:**

	*U*^11^	*U*^22^	*U*^33^	*U*^12^	*U*^13^	*U*^23^
Cl1	0.0508 (4)	0.0276 (3)	0.0642 (5)	−0.0062 (3)	0.0173 (3)	−0.0023 (3)
O1	0.0339 (9)	0.0328 (9)	0.0559 (11)	−0.0003 (7)	0.0190 (8)	−0.0044 (8)
O2	0.0438 (12)	0.0814 (16)	0.0696 (15)	0.0176 (11)	0.0135 (10)	0.0081 (12)
O3	0.0353 (9)	0.0315 (9)	0.0628 (12)	−0.0071 (7)	0.0207 (9)	−0.0071 (8)
O4	0.0525 (12)	0.0327 (10)	0.0694 (14)	−0.0093 (9)	0.0145 (10)	−0.0046 (9)
N1	0.0373 (12)	0.0223 (10)	0.0611 (14)	−0.0008 (9)	0.0158 (10)	−0.0023 (10)
N2	0.0325 (11)	0.0270 (11)	0.0661 (15)	−0.0004 (9)	0.0181 (10)	0.0036 (10)
N3	0.0276 (10)	0.0264 (10)	0.0527 (13)	−0.0003 (8)	0.0127 (9)	0.0009 (9)
C1	0.0330 (14)	0.0291 (13)	0.0715 (19)	−0.0040 (11)	0.0184 (13)	0.0048 (12)
C2	0.0279 (12)	0.0266 (12)	0.0412 (14)	0.0007 (9)	0.0083 (10)	0.0030 (10)
C3	0.0280 (12)	0.0289 (12)	0.0361 (13)	0.0010 (9)	0.0073 (10)	0.0027 (10)
C4	0.0311 (12)	0.0232 (11)	0.0424 (14)	0.0007 (9)	0.0091 (10)	0.0008 (10)
C5	0.0299 (12)	0.0299 (12)	0.0377 (13)	−0.0032 (10)	0.0080 (10)	0.0028 (10)
C6	0.0297 (12)	0.0329 (13)	0.0361 (13)	0.0023 (10)	0.0095 (10)	0.0009 (10)
C7	0.0361 (13)	0.0237 (11)	0.0447 (14)	0.0023 (10)	0.0107 (11)	−0.0030 (10)
C8	0.0299 (12)	0.0263 (12)	0.0394 (13)	−0.0007 (9)	0.0068 (10)	0.0027 (10)
C9	0.0431 (15)	0.0349 (14)	0.0490 (16)	0.0055 (12)	0.0147 (12)	−0.0035 (12)
C10	0.0493 (16)	0.0390 (15)	0.0512 (16)	0.0085 (12)	0.0221 (13)	0.0014 (13)
C11	0.045 (2)	0.152 (4)	0.135 (4)	0.034 (2)	0.019 (2)	−0.013 (4)
C12	0.0350 (14)	0.0305 (13)	0.0636 (18)	−0.0034 (11)	0.0162 (12)	−0.0027 (12)
C13	0.0448 (16)	0.0404 (15)	0.073 (2)	−0.0095 (12)	0.0240 (15)	−0.0085 (14)
C14	0.093 (3)	0.066 (2)	0.077 (3)	−0.032 (2)	0.031 (2)	−0.0167 (19)
C15	0.0338 (13)	0.0260 (12)	0.0403 (14)	0.0051 (10)	0.0125 (11)	0.0077 (10)
C16	0.0385 (14)	0.0353 (14)	0.0443 (15)	0.0027 (11)	0.0079 (11)	0.0015 (11)
C17	0.0542 (17)	0.0441 (16)	0.0466 (16)	0.0070 (13)	0.0167 (13)	−0.0070 (13)
C18	0.0421 (16)	0.0517 (17)	0.0538 (17)	0.0127 (13)	0.0230 (13)	0.0022 (14)
C19	0.0330 (13)	0.0352 (14)	0.0533 (17)	0.0038 (11)	0.0158 (12)	0.0107 (12)
C20	0.0346 (13)	0.0285 (12)	0.0496 (16)	0.0018 (10)	0.0123 (11)	−0.0001 (11)
C21	0.0387 (16)	0.0495 (17)	0.064 (2)	0.0052 (13)	0.0237 (14)	0.0062 (14)
C22	0.0416 (19)	0.077 (2)	0.082 (2)	−0.0098 (17)	0.0233 (17)	−0.0084 (19)

### Geometric parameters (Å, °)

**Table:**

O1—C6	1.352 (3)	C9—H9B	0.9700
O1—C9	1.441 (3)	C10—H10A	0.9700
O2—C10	1.397 (3)	C10—H10B	0.9700
O2—C11	1.419 (4)	C11—H11A	0.9600
O3—C5	1.361 (3)	C11—H11B	0.9600
O3—C12	1.436 (3)	C11—H11C	0.9600
O4—C13	1.412 (3)	C12—C13	1.507 (3)
O4—C14	1.412 (4)	C12—H12A	0.9700
N1—C1	1.317 (3)	C12—H12B	0.9700
N1—C8	1.378 (3)	C13—H13A	0.9700
N1—HN1	0.8600	C13—H13B	0.9700
N2—C1	1.309 (3)	C14—H14A	0.9600
N2—C2	1.353 (3)	C14—H14B	0.9600
N3—C2	1.337 (3)	C14—H14C	0.9600
N3—C15	1.427 (3)	C15—C16	1.388 (3)
N3—H3	0.8600	C15—C20	1.389 (3)
C1—H1	0.9300	C16—C17	1.382 (4)
C2—C3	1.440 (3)	C16—H16	0.9300
C3—C8	1.400 (3)	C17—C18	1.375 (4)
C3—C4	1.416 (3)	C17—H17	0.9300
C4—C5	1.368 (3)	C18—C19	1.386 (4)
C4—H4	0.9300	C18—H18	0.9300
C5—C6	1.429 (3)	C19—C20	1.399 (3)
C6—C7	1.370 (3)	C19—C21	1.444 (4)
C7—C8	1.397 (3)	C20—H20	0.9300
C7—H7	0.9300	C21—C22	1.170 (4)
C9—C10	1.491 (3)	C22—H22	0.95 (3)
C9—H9A	0.9700		
			
C6—O1—C9	117.09 (19)	H10A—C10—H10B	108.3
C10—O2—C11	111.7 (3)	O2—C11—H11A	109.5
C5—O3—C12	116.48 (18)	O2—C11—H11B	109.5
C13—O4—C14	112.8 (2)	H11A—C11—H11B	109.5
C1—N1—C8	120.4 (2)	O2—C11—H11C	109.5
C1—N1—HN1	119.8	H11A—C11—H11C	109.5
C8—N1—HN1	119.8	H11B—C11—H11C	109.5
C1—N2—C2	117.6 (2)	O3—C12—C13	106.5 (2)
C2—N3—C15	127.4 (2)	O3—C12—H12A	110.4
C2—N3—H3	116.3	C13—C12—H12A	110.4
C15—N3—H3	116.3	O3—C12—H12B	110.4
N2—C1—N1	125.4 (2)	C13—C12—H12B	110.4
N2—C1—H1	117.3	H12A—C12—H12B	108.6
N1—C1—H1	117.3	O4—C13—C12	111.1 (2)
N3—C2—N2	117.4 (2)	O4—C13—H13A	109.4
N3—C2—C3	121.2 (2)	C12—C13—H13A	109.4
N2—C2—C3	121.4 (2)	O4—C13—H13B	109.4
C8—C3—C4	117.6 (2)	C12—C13—H13B	109.4
C8—C3—C2	116.7 (2)	H13A—C13—H13B	108.0
C4—C3—C2	125.7 (2)	O4—C14—H14A	109.5
C5—C4—C3	120.7 (2)	O4—C14—H14B	109.5
C5—C4—H4	119.7	H14A—C14—H14B	109.5
C3—C4—H4	119.7	O4—C14—H14C	109.5
O3—C5—C4	125.2 (2)	H14A—C14—H14C	109.5
O3—C5—C6	114.5 (2)	H14B—C14—H14C	109.5
C4—C5—C6	120.3 (2)	C16—C15—C20	120.1 (2)
O1—C6—C7	124.4 (2)	C16—C15—N3	117.1 (2)
O1—C6—C5	115.7 (2)	C20—C15—N3	122.8 (2)
C7—C6—C5	119.9 (2)	C17—C16—C15	119.7 (2)
C6—C7—C8	119.2 (2)	C17—C16—H16	120.1
C6—C7—H7	120.4	C15—C16—H16	120.1
C8—C7—H7	120.4	C18—C17—C16	120.6 (3)
N1—C8—C7	119.4 (2)	C18—C17—H17	119.7
N1—C8—C3	118.3 (2)	C16—C17—H17	119.7
C7—C8—C3	122.2 (2)	C17—C18—C19	120.2 (2)
O1—C9—C10	108.1 (2)	C17—C18—H18	119.9
O1—C9—H9A	110.1	C19—C18—H18	119.9
C10—C9—H9A	110.1	C18—C19—C20	119.6 (2)
O1—C9—H9B	110.1	C18—C19—C21	120.0 (2)
C10—C9—H9B	110.1	C20—C19—C21	120.4 (2)
H9A—C9—H9B	108.4	C15—C20—C19	119.6 (2)
O2—C10—C9	108.7 (2)	C15—C20—H20	120.2
O2—C10—H10A	109.9	C19—C20—H20	120.2
C9—C10—H10A	109.9	C22—C21—C19	177.3 (3)
O2—C10—H10B	109.9	C21—C22—H22	178 (2)
C9—C10—H10B	109.9		
			
C2—N2—C1—N1	4.2 (4)	C1—N1—C8—C3	−1.7 (4)
C8—N1—C1—N2	−2.7 (4)	C6—C7—C8—N1	177.9 (2)
C15—N3—C2—N2	1.5 (4)	C6—C7—C8—C3	−2.4 (4)
C15—N3—C2—C3	−178.7 (2)	C4—C3—C8—N1	−177.0 (2)
C1—N2—C2—N3	178.4 (2)	C2—C3—C8—N1	4.1 (3)
C1—N2—C2—C3	−1.3 (4)	C4—C3—C8—C7	3.4 (4)
N3—C2—C3—C8	177.5 (2)	C2—C3—C8—C7	−175.5 (2)
N2—C2—C3—C8	−2.7 (3)	C6—O1—C9—C10	176.3 (2)
N3—C2—C3—C4	−1.2 (4)	C11—O2—C10—C9	−177.2 (3)
N2—C2—C3—C4	178.5 (2)	O1—C9—C10—O2	−76.9 (3)
C8—C3—C4—C5	−1.2 (3)	C5—O3—C12—C13	−170.3 (2)
C2—C3—C4—C5	177.5 (2)	C14—O4—C13—C12	−79.6 (3)
C12—O3—C5—C4	−11.3 (3)	O3—C12—C13—O4	−179.3 (2)
C12—O3—C5—C6	168.7 (2)	C2—N3—C15—C16	−146.2 (2)
C3—C4—C5—O3	178.3 (2)	C2—N3—C15—C20	35.6 (4)
C3—C4—C5—C6	−1.7 (4)	C20—C15—C16—C17	−1.7 (4)
C9—O1—C6—C7	−8.6 (3)	N3—C15—C16—C17	−179.9 (2)
C9—O1—C6—C5	172.4 (2)	C15—C16—C17—C18	−0.1 (4)
O3—C5—C6—O1	1.8 (3)	C16—C17—C18—C19	1.1 (4)
C4—C5—C6—O1	−178.3 (2)	C17—C18—C19—C20	−0.5 (4)
O3—C5—C6—C7	−177.3 (2)	C17—C18—C19—C21	179.5 (3)
C4—C5—C6—C7	2.7 (4)	C16—C15—C20—C19	2.4 (4)
O1—C6—C7—C8	−179.6 (2)	N3—C15—C20—C19	−179.6 (2)
C5—C6—C7—C8	−0.6 (4)	C18—C19—C20—C15	−1.3 (4)
C1—N1—C8—C7	177.9 (2)	C21—C19—C20—C15	178.7 (2)

### Hydrogen-bond geometry (Å, °)

**Table:**

*D*—H···*A*	*D*—H	H···*A*	*D*···*A*	*D*—H···*A*
N3—H3···Cl1	0.86	2.46	3.277 (2)	160
N1—HN1···Cl1^i^	0.86	2.23	3.066 (2)	165
C1—H1···O4^i^	0.93	2.46	3.372 (3)	167

Symmetry codes: (i) −*x*+1, *y*+1/2, −*z*+3/2.
